# The Extracellular Matrix in Epithelial Ovarian Cancer – A Piece of a Puzzle

**DOI:** 10.3389/fonc.2015.00245

**Published:** 2015-11-02

**Authors:** Angela Cho, Viive M. Howell, Emily K. Colvin

**Affiliations:** ^1^School of Medical and Molecular Biosciences, University of Technology Sydney, Sydney, NSW, Australia; ^2^Bill Walsh Translational Cancer Research Laboratory, Kolling Institute, Northern Sydney Local Health District, St. Leonards, NSW, Australia; ^3^Sydney Medical School Northern, University of Sydney, Sydney, NSW, Australia

**Keywords:** extracellular matrix, ovarian cancer, proteases, collagen, proteoglycan

## Abstract

Epithelial ovarian cancer is the fifth leading cause of cancer-related deaths in women and the most lethal gynecological malignancy. Extracellular matrix (ECM) is an integral component of both the normal and tumor microenvironment. ECM composition varies between tissues and is crucial for maintaining normal function and homeostasis. Dysregulation and aberrant deposition or loss of ECM components is implicated in ovarian cancer progression. The mechanisms by which tumor cells induce ECM remodeling to promote a malignant phenotype are yet to be elucidated. A thorough understanding of the role of the ECM in ovarian cancer is needed for the development of effective biomarkers and new therapies.

## Introduction

Epithelial ovarian cancer (EOC) is currently the most lethal gynecological malignancy affecting women and the fifth leading cause of cancer-related deaths in the United States (1). Early diagnosis of EOC grants a favorable prognosis and an average 5-year survival rate of 92%. However, due to the lack of available screening tests (2), diagnosis of patients is predominantly made at an advanced stage, reducing the average 5-year survival rate to only 27% (3). Standard treatment has not significantly improved for decades. The tumor microenvironment is gaining recognition in facilitating cancer progression, playing an essential role in mediating the growth, invasion, and metastasis of malignant tumors and therefore represents an attractive therapeutic target in solid tumors, including EOC. The tumor microenvironment consists of a variety of cell types including fibroblasts, immune cells, and endothelial cells, as well as non-cellular components such as the extracellular matrix (ECM), ECM remodeling enzymes [e.g., matrix metalloproteinases (MMPs), tissue inhibitors of metalloproteinases (TIMPs), and lysyl oxidases (LOXs)], and growth factors (e.g., VEGF, TGF-β, and PDGF). All these components work to create a microenvironment permissive for tumor cell growth, migration, and invasion. This review will focus on our current understanding of the roles that the ECM and ECM remodeling enzymes play in EOC progression, with specific emphasis placed on the individual key factors in the ECM known to date.

### ECM Remodeling Promotes Ovarian Cancer Progression

The ECM is constructed from cellular secretions and is a critical regulator of normal tissue development and function (4). It is a dynamic, non-cellular structure existing within all tissues, which not only serves as a physical support for cells, but also has a unique role in tissue homeostasis (5). These diverse functions of the ECM are conferred through its complex organization, composition, and its continuous remodeling. The constituents of the ECM in different tissues vary, imparting a unique ability to accommodate the specific needs required by different tissues (6). This is facilitated by the chemical and physical interactions between the resident cells and the continuously changing microenvironment (7). The ECM is composed of two main types of macromolecules: fibrous proteins and proteoglycans (8).

**Figure 1:**
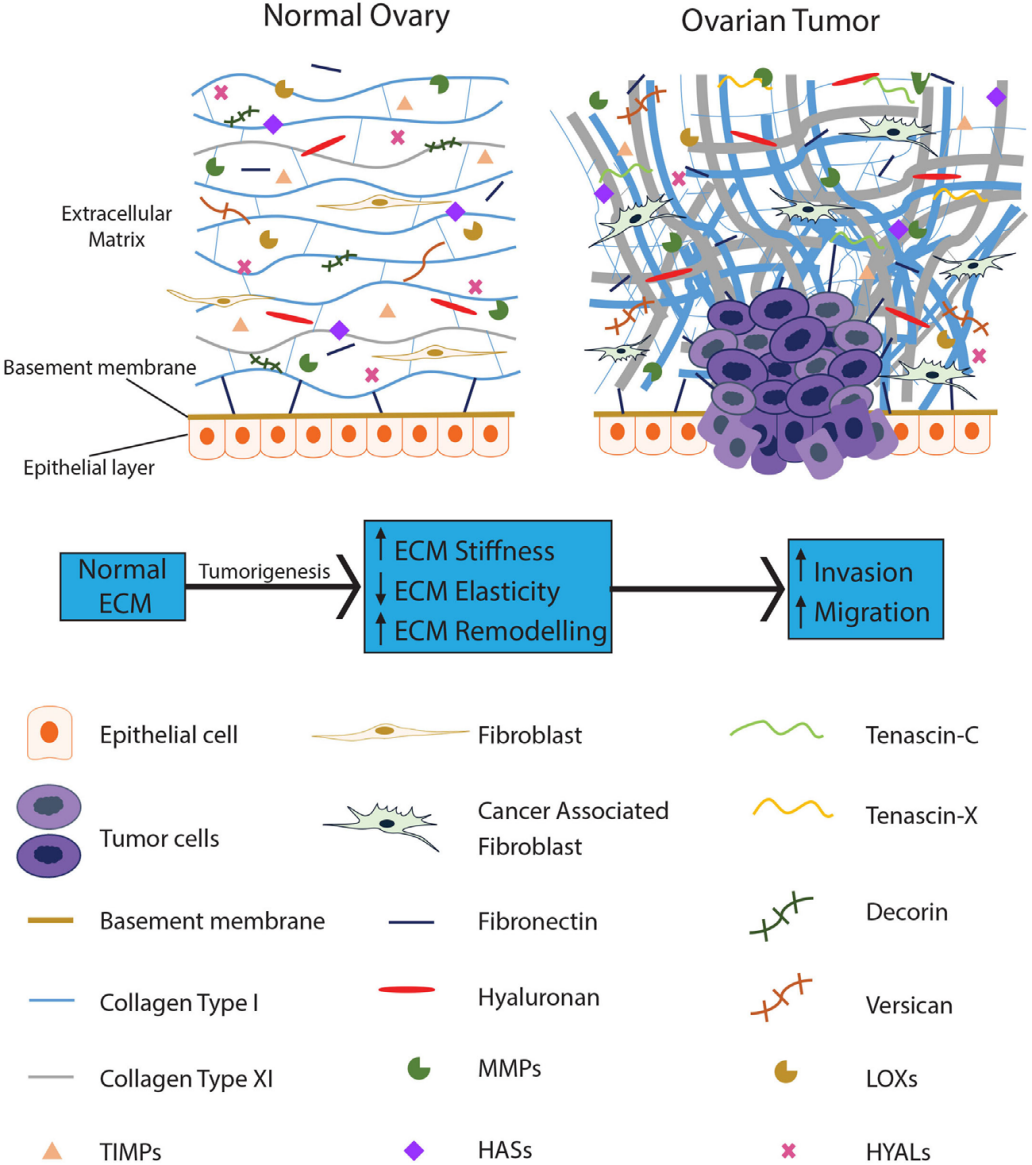
**The ECM becomes dysregulated during ovarian tumorigenesis and contributes to tumor progression**. The normal ovarian ECM consists of a highly ordered arrangement of collagen fibers, with hyaluronan interspersed throughout, regulating the distribution of the collagen in the ECM. Several proteoglycans, such as decorin and versican, are present to provide pressure and hydration to the tissue. In EOC, stromal fibroblasts are activated; collagen becomes progressively remodeled into short thick fibrils, randomly orientated into tracks at angles tending toward perpendicular rather than parallel to the epithelial boundary. In addition, versican, fibronectin, tenascin-C, and tenascin-X are upregulated with the loss of decorin. Reduced levels of HYALs lead to accumulation of hyaluronan; upregulation of LOXs leads to increased crosslinking of the ECM proteins resulting in increased stiffness of the ECM. MMPs are overexpressed in the EOC ECM, actively remodeling the ECM to promote tumor progression while TIMPs are unable to restrain these enzymes from dysregulating the ECM.

An increasing number of studies have proposed an essential role for the ECM in tumor progression, with dysregulation of the ECM implicated in cancer and characterized by extensive modification of its structure and composition. The secretion and/or inhibition of various ECM components and the subsequent remodeling by tumor cells creates a protumorigenic microenvironment which ultimately assists in tumor cell survival while disregarding the normal physiological function of the tissue (9). Stiffness and atypical ECM deposition are recognized in various cancers (10), with ECM alteration necessary for tumor initiation, progression, and intraperitoneal dissemination in EOC (11). Figure 1 provides a schematic representation of the ECM components involved in EOC.

## Fibrous Proteins

Fibrous proteins are major components of the ECM that provide tensile strength, elasticity, and structure to tissues. Many of these proteins become dysregulated in solid tumors and contribute to tumor growth and metastasis. Listed below are the fibrous proteins involved in EOC development and progression. Table 1 provides a summary of the ECM fibrous proteins reviewed here and their potential roles in EOC.

**Table 1:** **Summary of the ECM proteins and their roles in EOC**.

	ECM protein	Role in EOC	Reference
Fibrous proteins	Collagen I	Preferential and strong adhesion of primary ovarian cancer cells and spheroids to collagen I	(12, 13)
		Promotes migration	(14, 15)
		Provides a steering cue for cell migration	(16)
	Collagen XI	Expression levels correlate with tumor grade	(17–19)
		Associated with poor clinical outcome and overall survival	(20, 21)
		Predictor of recurrence	(17)
		Contributes to paclitaxel resistance by upregulating tau	(22)
	Fibronectin	Expressed in the ECM and ascites	(23, 24)
		Indicator of poor prognosis	(25)
		Mediates migration, invasion, and metastasis	(15, 23, 26, 27)
		Fibronectin fragments enhances adhesion of EOC cells to the peritoneal surface	(28)
	Tenascin-C	Promoted increased adhesion and migration	(29)
	Tenascin-X	Levels associated with tumor grade	(30)
		Strong positive correlation with serum CA-125 levels	(31)
	Laminin	Absent in microinvasive cells and low malignant tumors	(32)
		Significantly higher in EOC ascites than normal peritoneal fluid	
		No difference in serum levels between EOC and healthy control	
		Significantly higher serum levels in malignant EOC than in benign tumors and healthy controls	(33)
		Ascites levels >serum levels in malignant EOC	
		Serum levels significantly reduced after surgery	(34)
Proteoglycans	Decorin	Cancer progression associated with reduced or loss of expression in EOC ECM	(35–38)
	Lumican	Downregulation may have role in cancer aggression	(39)
	Versican	Elevated levels in EOC ECM correlated with poor disease outcome	(40–42)
	Perlecan	Expression lost in BM which facilitated invasion	(43)
	Hyaluronan	Elevated HA levels correlated with tumor grade and metastasis	(44)
		Strong, independent prognostic factor	(42)
		Positive correlation with invasion and metastasis	(42, 45)
		Facilitates adhesion of tumor cells to the peritoneum	(11, 46, 47)
		Reduces efficacy of chemotherapy and induces chemoresistance in response to chemotherapy	(48)
		Conjugates with chemotherapy increased the efficacy of chemotherapy	(49, 50)

### Collagens

Collagens are the most abundant fibrous proteins of the ECM. They associate with other collagens and interact with extracellular proteins, glycosaminoglycans and nucleic acids (7). Currently, there are 28 types of collagen identified which are divided into three major sub-groups: fibrillar, non-fibril forming, and fibril-associated collagens (51–53). Fibrillar collagens are the predominant sub-group present in the ECM, and their unique functions are governed by their conformation and structure, allowing them to form highly organized fibrils (6, 51). Particular focus will be placed on this specific sub-family of collagens. Fibrillar collagens are comprised of three α chains which assemble intracellularly into a triple helix that is secreted into the ECM as a procollagen molecule. Cleavage by metalloproteinases present in the ECM convert procollagen into collagen (6).

Insight into collagen morphology and organization and the resultant fibril composition and structure in the ECM are crucial for understanding how structural modifications are associated not only with the normal physiology of healthy tissue, but also with malignant processes linked to cancer progression (54). In normal tissue including normal ovary, collagen is organized as thin, long wavy fibrils, parallel to the epithelial boundary and providing elasticity to the ECM (Figure 1). In contrast, collagen remodeling in tumor stroma results in thicker and shorter fibrils, bunched into tracts at angles tending toward perpendicular to the epithelial boundary (Figure 1). Collagen tracts perpendicular to the epithelial boundary (also known as Tumor-Associated Collagen Signature (TACS)-3) are found in EOC (55). TACS-3 may facilitate entry into the stroma with invasive foci observed at these sites in mammary tumors (56). TACS-3 is also associated with a loss of elasticity and increase in stiffness of the ECM. These findings illustrate that ECM remodeling occurs in tumors and that fibrillar collagen contributes to this remodeling affecting the function of the resultant ECM.

Collagen-rich ECM was originally postulated to regulate normal tissue architecture and act as a physical barrier to tumor cell migration. However, it was shown that collagen-dense ECM induced by tumor cells essentially increased invasiveness and promoted tumor progression rather than inhibiting it (57). Increased risk of breast cancer has been associated with excessive collagen deposition and crosslinking (57, 58). Elevated collagen deposition and remodeling compromises drug delivery (59) and has been observed to be linked to cisplastin resistance in EOC (60–62). Collagen composition of the tumor ECM is crucial in mediating EOC progression and contributes to the poor response of ovarian cancer patients to chemotherapy, further emphasizing the importance of the ECM as an active participant in tumor progression.

#### Collagen I

Fibrillar type I collagen is the most abundant structural component of the ovarian ECM. Several *in vitro* studies have established the importance of type I collagen in EOC adhesion and migration, successfully demonstrating that collagen I enhanced migration of multiple EOC cell lines (14, 15), and primary EOC cells and spheroids preferentially and strongly adhered to type I collagen (12, 13). Collagen I has been demonstrated to have a novel role in inducing chemoresistance by upregulating tau (22), a microtubule-associated protein, which has been associated with paclitaxel resistance in EOC (63, 64). Flate and Stalvey (16) presented a study which implicates type I collagen as a steering mechanism for selected EOC cell lines *in vitro* and indicated that the migration of EOC cells induced by type I collagen was partially due to increased directionality. The promigratory cues which type I collagen confers on EOC cells highlight the multiple ways in which collagen can facilitate cancer cell migration. Thus, not only does collagen have a physical role in cancer progression, but it also has a potential role as a chemoattractant and may have an underlying role in chemoresistance. However, further studies are needed to consolidate these findings.

#### Collagen XI

Collagen XI is a minor fibrillary collagen predominantly found in cartilage with low or absent expression in most tissues (65–68). Hence stromal changes of collagen XI α-1 (COL11A1) expression are regarded as markers of cancer initiation and progression (20). High COL11A1 expression is associated with poor overall survival, poor clinical outcome and is a predictor of EOC recurrence correlating with the stage of disease (17, 20, 21). Increased gene expression of *COL11A1* was observed in all EOC patients during tumor progression and was greatly increased in metastases (18). Varying mRNA and protein expression levels of COL11A1 at different stages and sites of the tumor suggests COL11A1 as a potential biomarker, with the highest COL11A1 levels detected in late stage disease (recurrent metastases) and lowest levels in earlier stage disease (primary ovarian tumors) (19). Though COL11A1 is clearly associated with cancer progression and metastasis, there are a limited number of studies detailing the role and mechanism of COL11A1 overexpression in metastasis. With limited biomarkers available for EOC, COL11A1 has potential as a clinical screening tool and prognostic marker.

### Fibronectin

Fibronectin is implicated in cell growth, migration, and differentiation in processes including wound healing, embryonic development, and tumorigenesis (69, 70). Fibronectin plays a significant role in tumor progression, promoting metastasis, angiogenesis (71), and inhibiting apoptosis (72). Fibronectin expression is observed in the submesothelial basement membrane (BM) of metastatic omental tumors, ECM (23), and ascites (24). It is an indicator of poor prognosis in invasive EOC (25) and has been shown to mediate EOC cell migration and invasion (26) through the upregulation of the FAK/PI3K/Akt pathway (15). EOC cell motility and early metastatic competence is stimulated through the release of fibronectin from peritoneal mesothelial cells (23, 27). The protumorigenic role of fibronectin is further illustrated by Kenny et al. (23), who showed a significant reduction in the invasive and metastatic ability of EOC cells when fibronectin was knocked out from the peritoneal microenvironment. Another study by Kenny et al. (28) demonstrated that adhesion of EOC cells to the peritoneal surface was enhanced by MMP2 cleavage of fibronectin into small fragments. These studies have established fibronectin as a critical promoter of EOC migration and invasion. With its strong correlation with EOC progression, fibronectin presents a favorable target in cancer treatment.

### Tenascin

There are four large extracellular glycoproteins which constitute the tenascin family: –C, –X, –R, and –W (73). Tenascins have roles in cell adhesion and proliferation. In certain cell types, they act as antiproliferative agents, while in other cell types, they act to promote adhesion and migration (74).

#### Tenascin-C

Tenascin-C (TNC) is an important tissue remodeling glycoprotein which contributes to tumorigenesis and metastasis by promoting proliferation, invasion, and angiogenesis (29, 75). TNC is either absent or present in minute amounts in healthy, developed tissues and significantly increased in pathological conditions, such as cancer (75). High TNC expression has been demonstrated in solid tumors, including breast, pancreas, prostate, brain, and ovary. High TNC expression correlated with poor survival in lung, glioma, breast, and colon cancers (76). In EOC, TNC levels were significantly higher than in non-cancer controls (75) and increased with increasing grade and stage, with malignant tumors displaying the highest expression (30). A subsequent study by the same investigators demonstrated a 100-fold increase in ovarian fibroblast media compared to media derived from EOC cell lines, suggesting that TNC is predominately secreted by fibroblasts (29). This study also indicated a potential role of TNC in invasion, demonstrating increased adhesion and migration *in vitro*.

The consistent finding of increasing TNC levels with increasing tumor stage for several cancer types suggests a potential biomarker role for TNC. However, a study by Didem et al. (75) determined that serum TNC levels had no prognostic value in EOC, with no correlation between high serum TNC levels and any prognostic factors, including tumor stage and grade, response to chemotherapy or survival, although patients with high TNC levels were observed to have poorer overall survival (75). This study only investigated serum TNC levels as a prognostic marker. It did not examine TNC levels in the immediate ECM of the ovarian tumor. There are limited studies available which examine TNC in EOC tumor tissue specifically, and further studies are required to establish its potential role in EOC progression.

#### Tenascin-X

Tenascin-X (TNX) is the largest member of the tenascin family, and during development TNX is widely expressed (77). TNX levels are significantly elevated in EOC compared to healthy tissues, normal ovaries, and benign tumors (31). Levels in ascites from EOC patients correlated with serum CA-125, implying that TNX secretion may be coordinated with the release of CA-125 (31), and may prove useful as a potential biomarker to complement CA-125. However, like TNC, there are limited studies available implicating TNX in EOC development (30).

### Elastin

Elastin is a strong and insoluble biopolymer which constitutes ~90% of elastic fibers and is responsible for the resilience and elastic recoil of elastic vertebrate tissues (78). It has an extremely low turnover rate (79) and is formed through the crosslinking of its soluble precursor, tropoelastin, by LOX (80). Elastin has to some degree been associated with tumor growth and progression in other tumor types (81–83). Only one study is available to our knowledge which associates elastin with EOC. This study by Stewart et al. (84) evaluated the value of elastin staining in grading peritoneal implants associated with borderline serous EOC and demonstrated potential value in confirming superficial distribution of non-invasive peritoneal implants. However, this study did not specifically look at elastin levels in primary EOC tumors, and no studies are available at present which detail the potential role of elastin in EOC development and progression.

### Laminin

The laminin family of glycoproteins consists of 12 unique heterotrimers and is the major non-collagen structural component of the BM (85). In addition to its structural functions, laminin also regulates cell adhesion and migration, demonstrating a role in tissue homeostasis and morphogenesis (86–89). This is partially mediated by the interactions between laminin and other ECM molecules, such as collagen type IV, fibronectin, and heparin sulfate proteoglycans (90). The role of laminin in the BM of EOC has been well studied; however, its potential role in the ECM has not been thoroughly examined. Laminin was observed to be absent around microinvasive cells and also in tumors of low malignant potential in the early stages of invasion (32). Studies show elevated laminin levels in the ascites from EOC patients compared to normal peritoneal fluid; however, there are conflicting results as to whether this corresponds to an increase in serum laminin levels (33, 34). Though there are strong indications of the possible tumor-promoting roles of laminin in EOC, further studies are needed to establish this association and its potential value as a biomarker or therapeutic target.

## Proteoglycans

Proteoglycans are dispersed throughout ECM and act to provide compressive resistance and hydration to the tissue (6, 8). The two major ECM proteoglycan families consist of those containing leucine-rich repeats and hyalectans. Table 1 provides a summary of proteoglycans reviewed in this section and their potential roles in EOC.

### Small Leucine-Rich Repeat Proteoglycans

Leucine-rich repeat (LRR) proteoglycans are the most abundant and also the largest class of proteoglycans in the ECM. They have various functions, combining roles as signaling molecules and structural components during tissue remodeling in cancer and inflammation. LRR proteoglycans are regulated by the TGF-β and Wnt signaling pathways and interact with a range of Toll-like receptors, receptor tyrosine kinases, and growth factors to regulate homeostatic processes, such as apoptosis, migration, proliferation, angiogenesis, differentiation, and survival (91–96). They are also involved with regulating fibrillary collagen assembly, degradation, and organization (97–103). Of the LRR proteoglycan family members, only decorin and lumican have to date been shown to play roles in the ovarian tumor ECM.

#### Decorin

Decorin, a fundamental component of the ECM, binds to collagen and facilitates tissue scaffolding (104). However, its expression in cancer, including EOC, is generally reduced or undetectable (35–38, 105–107). Decorin-induced growth suppression was observed in a study by Merle et al. (108), highlighting the importance of decorin in possibly inhibiting tumor growth. It was proposed that decorin was able to interfere with the interactions between the resident cells and the ECM, by inhibiting fibronectin binding and integrin interaction. Nash et al. (109) and Teicher et al. (110) demonstrated the synergistic effects of decorin with cisplastin and carboplatin in inhibiting the growth of breast and EOC. Decorin can inhibit tumor growth by suppressing TGF-β (105, 111) and directly interacting with the epidermal growth factor receptor and ERBB2 (112–115). The direct interaction with these receptors diminishes receptor-mediated intracellular signaling and induces apoptosis (116, 117). These studies suggest that decorin plays a major role in controlling tumor growth and its subsequent downregulation is associated with EOC development, indicating that possible therapies involving the restoration of decorin expression in the tumor stroma, coupled with chemotherapy, could potentially retard the growth of EOC.

#### Lumican

Lumican, another LRR proteoglycan, is involved in the regulation of collagen fibrillogenesis, migration, invasion, angiogenesis, and apoptosis (104, 118–120). Varying levels have been reported in the stroma of different tumor types (121–126). In breast and pancreatic cancers, high stromal lumican was associated with advanced cancer stage, invasion, and poor survival (127, 128), whereas a negative correlation was found between tumor grade and expression in neuroendocrine tumors of the colon (129). In support of this, lumican has been shown to inhibit tumor growth and progression in lung cancer and melanoma (92). To date, a single study has examined lumican expression in EOC, demonstrating reduced stromal expression and suggesting a possible role in cancer aggression (39). Given the varying clinical associations with lumican in several cancer types, more research is needed to determine the precise role of lumican in EOC.

### Versican

In the healthy ovary, versican (VCAN) is tightly regulated and acts as an important ECM proteoglycan present in the granulosa cells of growing follicles, to aid in the expansion of the cumulus oophorus in the preovulatory stage (130). Normal processes, such as wound healing (131), follicle growth (130), and inflammation (132), induce VCAN expression. Many malignant tumors have elevated levels of VCAN (133–140). Elevated VCAN levels were also observed in the ECM of EOC and correlated with increased hyaluronan (HA) levels, suggesting that they may form a supportive partnership to assist in EOC survival and spread (40, 41). *In vitro* studies have demonstrated the production of VCAN by malignant cells; however, the source of VCAN in tumors remains to be elucidated, with no clear answer on the *in vivo* source of VCAN, which may also include stromal cells (137, 141–143). Upregulation of VCAN in EOC correlates with a poor disease outcome; however, the significance of VCAN as a prognostic marker is debatable, with HA, its binding partner, presenting greater value as a prognostic marker (42). The same study showed that despite a strong association with the apparent initiation and development of EOC, VCAN was not an independent indicator for patient survival.

### Perlecan

Perlecan is a core heparan sulfate proteoglycan of the ECM and BMs of normal tissues and blood vessels (8, 144, 145). Perlecan is stored in abundant quantities in the BM. Degradation of the BM by MMPs during tumor invasion causes the release of perlecan into the ECM, and increased expression is reported in various cancer cell lines and tumors (146–148). Perlecan has been suggested to have various roles in tumor progression, by regulating the cell’s response to mitogenic and angiogenic growth factors, and mediating adhesion and migration (149, 150). Its inhibition is reported to impede tumor growth and invasion (144, 151). A study by Davies et al. (43) observed heterogeneous perlecan expression in ovarian tumors compared to normal ovary. Perlecan expression was observed in the BM, the stroma, the internal elastic lamina of blood vessels, and the submesothelial layer of the normal ovary. Perlecan was also present in the BM of benign and borderline tumors but absent in the BM of malignant tumors, enhancing invasive potential. Loss of perlecan was not observed in the stroma and BMs of blood vessels. Further studies are needed to determine its potential role in EOC progression.

### Hyaluronan

Hyaluronan is strongly implicated in cell proliferation, migration, wound healing, and inflammation (152). HA binds and interacts closely with fibronectin during matrix construction (152–154) and regulates the distribution of collagen fibrils (155). HA can interact directly with cells by binding to cell surface receptors and constructing a protective coat (156–158). It also has a role in the distribution of proteoglycans in the ECM through non-covalent interactions (159–161). Changes in HA content and size are associated with tissue remodeling and pathological processes, such as tumor progression (162–166). Studies from several cancer types have recognized the elevation of HA in serum (167–169), and recently a study by Wu et al. (170) demonstrated the novel use of serum HA in differentiating non-metastatic from metastatic breast cancer, which suggests HA as a potential biomarker. Elevated HA levels were observed in and associated with tumor aggression in breast, lung, prostate, colorectal, and bladder cancer (163).

Epithelial ovarian cancer grade and metastasis are correlated with increasing HA levels, with Hiltunen et al. (44) demonstrating a 100-fold increase in HA expression in grade three EOC. Many cancers, including ovarian, are enveloped in a HA rich ECM (11, 42, 44). HA upregulation has been implicated as a strong, independent prognostic factor for EOC (42) and is positively correlated with invasion and metastasis (42, 45). Adhesion of ovarian cancer cells to the peritoneum is facilitated by interactions between HA and its major surface receptor CD44 (45–47, 171). HA has been shown to reduce the ability of chemotherapeutic drugs to induce cell death in several cancers (172–175). Ricciardelli et al. (48) demonstrated chemotherapy-induced HA production facilitates chemoresistance and EOC cell survival through a HA-CD44-mediated pathway. HA-chemotherapy conjugates were successful in increasing the efficacy of standard chemotherapy in EOC patients by CD44-mediated uptake of the chemotherapy (49, 50). Hence, HA is a promising therapeutic target, with HA inhibition potentially suppressing adhesion of EOC cells to the peritoneum, which is the preferential place for EOC metastasis. Conversely, HA can be utilized to enhance the cytotoxic effects of chemotherapy.

## Enzymes

Abnormal expression and deposition of ECM components and alteration to its structure are implicated in malignancies such as EOC. Remodeling of the ECM in healthy tissues through chemical modification, synthesis, degradation, and reassembly are tightly controlled processes induced by cells in homeostasis (176). Crosstalk between the ECM and cancer cells causes the alteration of ECM structure and composition, resulting in the dysregulation of this tightly controlled system (5). Cleavage of ECM components by proteases and the subsequent remodeling of the ECM is implicated in EOC progression, where the degradation of the BM and ECM is necessary for the invasion and metastasis of EOC cells (177, 178). Malignant cells produce a wide range of ECM-degrading proteases implicated in ECM dysregulation and cancer progression. Inhibiting their activity may be of potential therapeutic value. Table 2 provides a summary of the ECM remodeling enzymes reviewed here and their potential roles in EOC.

**Table 2:** **Summary of the ECM remodeling enzymes and their roles in EOC**.

Enzyme	Role in EOC	References
MMP2/MMP9	Expression associated with EOC aggression	(179–181)
	Identified to be secreted by cancer cells and expression correlated with invasiveness	(182–185)
	Higher total activity in the metastatic site	
	Promotes metastasis	(180)
	MMP9 – conflicting reports as a prognostic marker	
	High levels of epithelial MMP9 associated with a better DRS	(179, 188)
	High stromal levels of MMP associated with worse DRS	(189–192)
	High epithelial and stromal MMP9 associated with poor DRS and metastasis	
MMP7	Conflicting findings in promoting tumor progression	
	Overexpression promoted invasion	(193)
	Suppression of MMP7 inhibited migration and invasion	(194)
	MMP7 expression lower in malignant tumors	(181, 195)
	Higher expression correlated with good clinical and survival parameters	(196)
LOX	LOX G473A polymorphism correlated with advanced stages and increased susceptibility	(197, 198)
	Overexpression correlated with metastasis and tumor stage	(199)
	Promoted migration and tumor growth by repressing E-cadherin	(200)
LOXL2	Upregulated specifically in EOC endothelial cells. Inhibition of LOXL2 reduced endothelial cell concentration	(201)
	LOXL2 inhibition suppressed tumor angiogenesis and induced normalization of tumor-associated vasculature	(202)
LOXL4	Tumor suppressive effect, however, LOXL4 splice variants enhanced tumor progression and metastatic potential	(203)
Hyaluronan Synthases	Low HAS1 – independent predictor of ovarian cancer patient survival High HAS1 correlated with high microvessel density in ovarian cancer	(204)
	*HAS2* and *HAS3* – no consistent increase	
	*HAS1* – barely detectable in EOC	(205)
	*HAS1*, *HAS2*, and *HAS3* – overexpressed in effusions, solid metastases, and primary EOC, respectively	(206)
	High *HAS1* expression in EOC effusion correlated with shorter survival	
Hyaluronidases	*HYAL1–3* – chromosomal loss in tumor and stromal tissue	(207)
	Reduced activity and expression in malignant EOC – also differentially expressed	(44, 206)
	*HYAL1* – absent in serous EOC	(206, 208)
	*HYAL2*-*var2* and *HYAL3* variant – overexpressed in solid metastases and primary EOC tumors, respectively	(206)

### Matrix Metalloproteinases

Matrix metalloproteinases are a family of extracellular proteins comprising >20 zinc metalloproteases which play major roles in tissue repair and remodeling in response to injury (209). In normal conditions, the activity of MMPs is low. However, in response to cellular and matrix interactions, growth factors, hormones, and inflammatory cytokines released during remodeling or repair processes and in inflamed or diseased tissues, MMP activity increases (5, 210). MMPs remodel the ECM and contribute to the tumor microenvironment by promoting tumor growth, metastasis, and angiogenesis (211, 212). Through these activities, MMPs promote cancer progression and correlate with poor patient prognosis (213).

#### MMP2 and MMP9

Matrix metalloproteinase 2 and MMP9 have been implicated to contribute to the malignant potential of tumor cells, due to their ability to degrade a major component of the BM, collagen type IV (214). MMP2 and MMP9 activity varies between normal ovaries and malignant ovarian tumors. MMP2 was observed to be prevalent in normal ovaries, while MMP9 was predominant in malignant tissues (215). EOC aggressiveness has been linked to MMP2 and MMP9 expression (179–181). Several studies have demonstrated the secretion of MMP2 and MMP9 from EOC cell lines *in vitro* and in ascites from advanced EOC patients. *In vitro* expression of MMP2 and -9 also correlated with the invasiveness of the EOC cell lines (182–185, 216). Schmalfeldt et al. (180) confirmed these studies, by not only demonstrating elevated levels of MMP2 and -9, but also identified higher total MMP2 and MMP9 activity in the metastatic site, compared to the primary site, suggesting their likely role in the progression from a benign state to an advanced stage. MMP2 expression correlated with clinical stage (186) and promoted metastasis along with MMP9 (187).

Sillanpää et al. (188) evaluated the prognostic significance of MMP9 in EOC, where high levels of epithelial MMP9 were associated with a better 10-year disease-related survival (DRS), while high stromal levels of MMP9 were associated with worse survival. This was supported by a study by Ozalp et al. (179). This however, has been contradicted by several other studies (189–192) which associated high epithelial and stromal MMP9 with shorter disease-specific survival and metastasis.

#### MMP7

Matrix metalloproteinase 7 is involved in the proteolysis of several ECM substrates, growth factors, and cellular receptors including collagens, proteoglycans, insulin-like growth factor-binding protein, heparin-binding epidermal growth factor, E-cadherin, and tumor necrosis factor-alpha precursor (193, 217). Overexpression of MMP7 in EOC has been demonstrated in several studies (193, 195, 196). Wang et al. (193) showed that overexpression of MMP7 promoted the invasion of EOC cells *in vitro* and likewise, suppression of MMP7 inhibited migration and invasion (194). These findings are in contrast to the study by Brun et al. (181) who showed that epithelial MMP7 expression was lower in malignant serous tumors, compared to its benign or borderline counterparts, while there was no difference observed among the mucinous tumors. Shigemasa et al. also supported this finding in mucinous ovarian tumors (195). MMP7 was observed to be an independent prognostic factor, with higher MMP7 expression in ovarian tumor cells correlating with good clinical and survival parameters (196). These discrepancies highlight the need to elucidate the functional role of MMP7 in EOC, where the grade and subtype of the tumor may result in these contrasting findings.

While MMP2, -7, and -9 have been implicated to play a role in EOC, there are discrepancies correlating the expression of these MMPs with the prognosis and certain clinicopathological features of EOC. This suggests that the functions of MMPs in EOC may be dependent on their epithelial or stromal associations in conjunction with the grade of the tumor and possibly the surrounding stroma. This highlights the complexity of MMPs and their roles in EOC progression and emphasizes the need for additional studies to provide an explanation for these differences. Nonetheless, MMPs have potential as therapeutic targets due to their indisputable activity during EOC progression. MMP2, -7, and -9 expression are elevated in EOC regardless of the grade; therefore, inhibition of MMPs may decrease the aggressiveness of EOC and aid in preventing invasion and metastasis.

### Tissue Inhibitors of Metalloproteinases

Tissue inhibitors of metalloproteinases are endogenous inhibitors of major ECM remodeling proteinases, such as MMPs, and subsequently they play a crucial role in regulating ECM composition and function. TIMPs also have multiple functions in regulating cell proliferation, migration, invasion, apoptosis, and angiogenesis (218). There are four TIMP paralogs: TIMP1–4 (219). TIMP1 is the most widely distributed TIMP and has the ability to inhibit all active forms of MMP (220). TIMP2 is most selective for MMP2 (221). Simultaneous increase of MMP2 and decrease of TIMP2 levels were observed in malignant tumors compared to benign tumors and the normal ovary, with this imbalance indicative of the importance of MMP2–TIMP2 levels in promoting invasion (222). In contrast, TIMP1 levels were increased in malignant tumors compared to the normal ovary (222–224). Okamoto et al. (222) observed that despite the synchronous increase of MMP9 and TIMP1 in malignant tumors, the degree of increase of MMP9 was much greater than TIMP1, suggesting that TIMP1 has limited ability in compensating for an increase in MMP9. A study by Kikkawa et al. (225) also reported elevated TIMP1 and MMP9 levels in EOC samples compared to normal ovary. Given their role as inhibitors of MMPs, the relative levels of each family of enzymes are important when considering their function in EOC. MMPs and TIMPs have a dynamic relationship not just in normal ovaries, but also in EOC, where the expression of TIMPs adjusts with the relative levels of MMPs present to ultimately promote tumor progression.

### Lysyl Oxidase

The LOX family comprises five members: LOX and four LOX-like isoenzymes LOXL1–4. LOX is a copper-dependent amine oxidase secreted by fibroblasts and, together with the other family members, has an important role in remodeling the ECM by regulating collagen and elastin crosslinking and therefore contributes to the strength and structure of many tissues (226–228). LOX and LOXL2 are heavily implicated in cancer progression (229, 230).

#### Lysyl Oxidase

The importance of the LOX family in ECM remodeling during normal physiological processes has been established in a variety of tissues. In the ovary, LOX is activated during ovulation, following follicle rupture and is critical in collagen synthesis and reassembly in the ovarian follicle (231). Several studies have demonstrated the expression of LOX in granulosa cells (232–234), and its expression and activity is tightly controlled by follicle-stimulating hormone during follicle development (232, 234). Wang et al. (197) initially showed that a single nucleotide polymorphism of the *LOX* gene, G473A, correlated with advanced stages and increased susceptibility to EOC in a Chinese population. This finding was supported by Wu et al. (198). In hypoxic conditions, hypoxia inducible factor-1α (HIF-1α) induced LOX expression and facilitated tumor migration, invasion, and metastasis in a range of cancers (229). LOX and HIF-1α overexpression were observed in hypoxic EOC cells, with expression levels correlating significantly with metastasis and tumor stage in EOC (199). HIF-1α upregulation induced LOX transcription through the accumulation of reactive oxygen species, subsequently repressing E-cadherin. Loss of E-cadherin was observed to promote EOC cell migration *in vitro* and tumor growth *in vivo*, while also correlating with tumor stage, differentiation, metastasis, and a poorer 5-year survival rate (200).

#### LOXL2

LOXL2 plays a similar role to LOX in crosslinking collagen and elastin in the ECM, contributing to the stability and strength of the tissue (226). LOXL2 overexpression has been linked to the aggressiveness of breast (235), skin (236), and colon cancers (237). Specific upregulation of the LOXL2 protein is found in EOC endothelial cells, where inhibition of LOXL2 reduced endothelial cell concentration within the tumor (201). A study by Zaffryar-Eilot et al. (202) confirmed the direct role of LOXL2 in angiogenesis, with LOXL2 inhibition decreasing microvessel density for the normalization of tumor-associated vasculature resulting in reduced tumor hypoxia with better response to therapy (238).

#### LOXL4

LOXL4 is expressed in head and neck squamous cell carcinoma and gastric cancer cell lines, and upregulation of LOXL4 significantly correlates with tumor stage and lymph node metastases (239–241). LOXL4 promotes proliferation, migration, and invasion in gastric cancer cell lines *in vitro* (241). A study by Sebban et al. (203) demonstrated a tumor suppressive effect in EOC *in vivo*, while also indicating a contrasting role of LOXL4 splice variants *in vitro*, with the variants enhancing tumor progression and metastatic potential. This study demonstrates the paradoxical roles of LOXL4 and its alternatively spliced isoforms. Specific variants of LOXL4 could be promising as a prognostic marker and a potential therapeutic target.

To our knowledge, only LOX, LOXL2, and LOXL4 have been studied in EOC. Aberrant LOX, LOXL2, and LOXL4 expression are implicated in dysregulating the ECM and inducing a malignant phenotype and promoting tumor progression in EOC. However, additional studies are needed to elucidate further potential roles of these LOX family members in EOC, where a wide range of studies have demonstrated a strong association between these enzymes and several tumorigenic pathways in a variety of other cancers (230). Due to the protumorigenic role of LOX, LOXL2, and LOXL4, inhibition of these LOX family members in conjunction with chemotherapy could potentially enhance its antitumorigenic effect and result in a better prognosis.

### Hyaluronan Synthases

Hyaluronan synthases (HASs) are integral plasma membrane proteins which synthesize HA (242, 243). Three isoenzymes of HAS with differing enzymatic activities have been identified in humans: HAS1, HAS2, and HAS3 (244). Overexpression of HAS is implicated to promote growth and metastasis in a variety of cancers through excessive production of HA (245–249). In one study of ovarian cancer by Yabushita et al., HAS1-negative tumors were associated with increased overall survival and lower microvessel density relative to HAS1-positive tumors. No relationships were found between the levels of HAS2–3 and tumor stage, survival, chemotherapy, or microvessel density (204). Weiss et al. (206) compared the expression of *HAS1–3* mRNA in serous EOC between effusions, primary carcinomas, and solid metastases, with differential *HAS* overexpression observed in each region. *HAS1*, *HAS2*, and *HAS3* were overexpressed in effusions, solid metastases, and primary carcinomas, respectively. High *HAS1* expression in EOC effusions correlated with shorter survival in agreement with the results of Yabushita et al. (204). However, a study by Nykopp et al. (205) demonstrated barely detectable *HAS1* in EOC and no consistent increase in the *HAS2* and *HAS3* expression. These result suggest unique roles for each HAS isoenzyme during different stages of EOC progression, although no clear associations have been identified to date.

### Hyaluronidases

Hyaluronan synthesis by HASs is opposed by the enzymatic action of hyaluronidases (HYALs), which degrade HA. The family of HYALs consists of six members (250), of which HYAL1 and HYAL2 are particularly well characterized (205). HYAL1 and HYAL2 are the main members responsible for HA turnover, with these two enzymes bearing several physiological and pathological roles, such as wound healing and inflammation (250, 251). High molecular weight HA is antiangiogenic; HYALs cleave HA into low molecular weight HA fragments which may promote angiogenesis, subsequently enhancing tumor growth (252). While chromosomal loss at the locus encoding *HYAL1–3* (3p21.3) is common in both tumor and stromal tissue from EOC patients, this allelic loss is not associated with increased tissue HA levels (207). HYAL activity and expression were reported to be reduced and differentially expressed in malignant EOC compared to its benign and normal counterparts (44, 206). *HYAL1* was absent in all serous EOC samples (206); however, expression of HYAL1 in EOC is subtype specific, with clear cell and mucinous EOC showing elevated levels of HYAL1 compared to serous and endometrioid EOC (208). Comparing transcript levels in serous EOC between primary tumors, solid metastases, and effusions, *HYAL*2 splice variant, *HYAL2-var2*, was significantly overexpressed in solid metastases, and *HYAL3 var1–3* was significantly underexpressed in solid metastases. A positive correlation was identified between *HYAL3* levels in effusions and paclitaxel treatment (206).

Regulation of HA synthesis and degradation is mediated by HASs and HYALs, respectively. As described above, HA accumulation is associated with the aggressiveness of EOC and has been demonstrated to promote EOC progression. The displacement of this otherwise delicate equilibrium of controlled HA synthesis and degradation by HASs and HYALs in EOC has major implications on the subsequent structure and function of the ECM and therefore may represent promising targets for cancer treatment.

## Discussion

The ECM is a dynamic structure. It is crucial in regulating specific function, development, and homeostasis, achieved by organizing and regulating the plethora of ECM components unique to each differentiated tissue (4, 7, 176, 253). Remodeling of the ECM in EOC is thought to promote tumor progression. The interactions between the ECM and the resident cells are tightly regulated, and disruption of the ECM has severe consequences as described in this review. It is evident that the ECM in EOC remains relatively unexplored, with the mechanisms involved in tumor progression yet to be fully elucidated. To fully understand how alterations to the ECM influence tumorigenesis, it is essential to investigate not only how the ECM interacts with tumor cells, but also how the ECM components interact with each other.

The constituents of the ECM offer potential biomarkers and therapeutic targets, where the manipulation of the ECM composition may complement current chemotherapeutic treatment. Enzymes involved in ECM remodeling and elevated in EOC, such as MMPs and LOXs, which have been shown in preclinical models to promote tumor progression in other cancers, could also be a potential therapeutic targets in EOC. Though CA-125 is clinically approved to be used as a serum tumor biomarker for ovarian cancer, Moss et al. (254) demonstrated its poor sensitivity and specificity, with a high false positive rate. Other than CA-125, there are currently no reliable biomarkers for the staging and prognosis of ovarian cancer. With low overall survival rates for patients diagnosed with advanced disease, a sensitive and specific diagnostic biomarker of early stage EOC is needed. Tenascins have a limited presence in healthy tissues, hence could potentially serve as biomarkers for early diagnosis of EOC.

Though the identification of individual ECM components allows us to understand their basic functions in EOC, the ECM must be considered not just as its individual elements, but as a collective entity. EOC progression is multifactorial and influenced by an altered ECM. The ECM constituents described in this review reflect the complexity of the EOC microenvironment which is an evolving area of research. Advances in understanding how the ECM contributes to EOC pathogenesis and progression will assist in the development of better treatments for EOC.

## Conflict of Interest Statement

The authors declare that the research was conducted in the absence of any commercial or financial relationships that could be construed as a potential conflict of interest.
